# Identification of Novel Sequence Types among *Staphylococcus haemolyticus* Isolated from Variety of Infections in India

**DOI:** 10.1371/journal.pone.0166193

**Published:** 2016-11-08

**Authors:** Sasmita Panda, Smrutiti Jena, Savitri Sharma, Benu Dhawan, Gopal Nath, Durg Vijai Singh

**Affiliations:** 1 Infectious Disease Biology, Institute of Life Sciences, Nalco Square, Bhubaneswar-751023, India; 2 Jhaveri Microbiology Centre, LV Prasad Eye Institute, Kallam Anji Reddy Campus, LV Prasad Marg, Banjara Hills, Hyderabad-500034, India; 3 Department of Microbiology, All India Institute of Medical Sciences, Ansari Nagar, New Delhi-110029, India; 4 Department of Microbiology, Institute of Medical Sciences, Banaras Hindu University, Varanasi-221005, India; Universitatsklinikum Munster, GERMANY

## Abstract

The aim of this study was to determine sequence types of 34 *S*. *haemolyticus* strains isolated from a variety of infections between 2013 and 2016 in India by MLST. The MEGA5.2 software was used to align and compare the nucleotide sequences. The advanced cluster analysis was performed to define the clonal complexes. MLST analysis showed 24 new sequence types (ST) among *S*. *haemolyticus* isolates, irrespective of sources and place of isolation. The finding of this study allowed to set up an MLST database on the PubMLST.org website using BIGSdb software and made available at http://pubmlst.org/shaemolyticus/. The data of this study thus suggest that MLST can be used to study population structure and diversity among *S*. *haemolyticus* isolates.

## Introduction

*Staphylococcus haemolyticus*, one of the coagulase-negative staphylococci (CoNS), is an opportunistic pathogen that ranks second after *S*. *epidermidis* in the frequency of isolation from blood cultures [1]. *S*. *haemolyticus* is notoriously known to exhibit multidrug resistance and form biofilm [2–8]. Multilocus sequence typing (MLST), a highly discriminatory and reproducible DNA-based typing technique has been utilized to analyze the bacterial isolates. The genetic analysis reported, at least, seventeen sequence types (STs) among *S*. *haemolyticus* strains, however, there is no MLST database in the public domain [3]. Therefore, we analyzed strains of *S*. *haemolyticus* isolated from a variety of infections collected from different parts of India and set up an MLST database at http://pubmlst.org/shaemolyticus/.

## Materials and Methods

### Ethics statement

The study was approved by Institutional Review Board (IRB) of LV Prasad Eye Institute (LEC/08/110/2009), and by Institute Ethics Sub-Committee (IESC) of All India Institute of Medical Sciences, New Delhi (IESC/T-34/2013), and the data were analyzed anonymously and reported.

### Bacterial isolates

A total of 34 *S*. *haemolyticus* isolates were included in this study. Seventeen isolates were from infected eyes, seven were from the healthy conjunctivae, six were from blood, and two isolates each were from pus and sputum, respectively. The isolates were identified by using Vitek 2 Compact System or Staph ID32 strips (bioMerieux, Marcy I’Etoile, France), 16S rDNA sequencing and amplification of *S*. *haemolyticus* specific *nuc* gene [9]. The strains were isolated from clinical samples sent to Microbiology Laboratories of LV Prasad Eye Institute, Bhubaneswar and Hyderabad, India, All India Institute of Medical Sciences, New Delhi, India, and Institute of Medical Sciences, Banaras Hindu University, Varanasi, India, respectively. The strains from asymptomatic healthy conjunctivae were isolated only after clinical examination by optometry and ophthalmology faculty that ruled out any ocular surface infection.

### Multilocus sequence typing and phylogenetic analysis

MLST of *S*. *haemolyticus* was performed using PCR product obtained with seven housekeeping genes [3]. Amplified product of seven housekeeping genes viz. *arc*, *SH1200*, *hemH*, *leuB*, *SH1431*, *cfxE*, and *RiboseABC* were purified (ExoSAP; Affymetrix, Cleveland, USA). Both the strands were sequenced using an ABI sequencer model 3500 (Life Technologies, Marsiling, Singapore) at the sequencing facility of the Institute of Life Sciences (Bhubaneswar, India). The nucleotide sequences were aligned using MEGA5.2 software and were manually compared with already reported alleles and STs. Sequencing was performed in biological duplicates to confirm the presence of novel alleles. New alleles were proposed and designated in comparison with the previously reported alleles [3]. A unique allele number was assigned to each sequence even if they differed at a single nucleotide site in a sequential manner and no weighting was applied to reflect the number of nucleotide differences between alleles. Subsequently, the novelty of new alleles was validated by Dr. Jorunn Pauline Cavanagh, Department of Pediatrics, University Hospital of North Norway (Personal communication).

The advanced cluster analysis was performed to define the clonal complexes (CC) by using Bionumerics software, version 7.1 (Applied Maths, Belgium). A minimum spanning tree (MST) was constructed using the MLST data and partitions were created to form clusters. The similarity in at least six alleles grouped isolates of *S*. *haemolyticus* in one CC. The central ST of each partition was used to designate a CC.

### MLST database for *S*. *haemolyticus*

The MLST database for *S*. *haemolyticus* was successfully set up at the website PubMLST.org using the BIGSdb software, and made available at http://pubmlst.org/shaemolyticus/ to assign STs or designate new STs among *S*. *haemolyticus* isolates [10].

### Nucleotide sequence accession numbers

The sequences of housekeeping genes were submitted to NCBI GenBank under accession numbers KM985504—KM985522 and KX073468—KX073483 for *arc*, KM985523—KM985541 and KX073484—KX073499 for *SH1200*, KM985542—KM985560 and KX073500—KX073515 for *hemH*, KM985561—KM985579 and KX073516—KX073531 for *leuB*, KM985580 -KM985598 and KX073532—KX073547 for *SH1431*, KM985599—KM985617 and KX073548—KX073563 for *cfxE*, KP019681—KP019699 and KX073564—KX073579 for *Ribose ABC*, respectively.

## Results and Discussion

**Fig 1:**
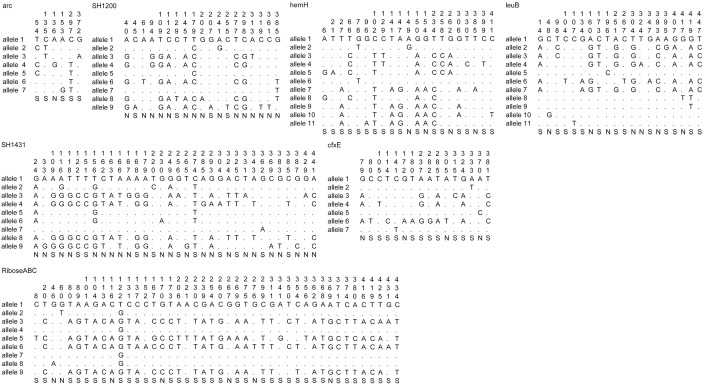
Polymorphic sites present in all loci among the *S*. *haemolyticus* isolates. For allele one, the nucleotide designation at the variable site is shown. For the rest of the alleles, only those sites that differ from allele one is shown. The position of the polymorphic site within the sequenced fragment is shown above (in vertical format), and synonymous (S) and non-synonymous (N) polymorphisms are shown below the figure.

**Fig 2:**
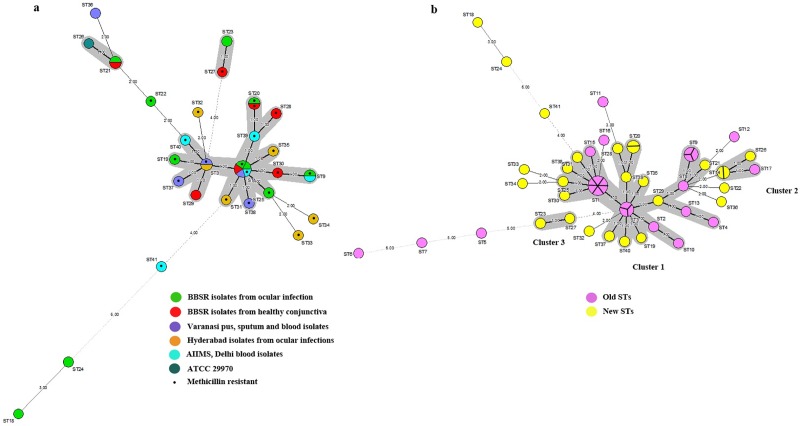
Minimum spanning tree generated using sequences of housekeeping genes consisting: *arc*, SH1200, *hemH*, *leuB*, SH1431, *cfxE* and *Ribose ABC*. **(A)** *S*. *haemolyticus* (34 isolates) collected during 2007–2016 from different places in India and reference *S*. *haemolyticus* strain ATCC 29970, **(B)** Combination of strains includes previously reported STs (ST1-ST17; pink circles) and new STs (ST18-ST41; yellow circles) found in this study. Minimum difference in two alleles separates the nodes into different clusters.

MLST analysis of *S*. *haemolyticus* showed that the number of polymorphic nucleotide sites at the seven loci varied from 6 (*arc*) to 44 (*Ribose ABC*) (Fig 1). The number of alleles present at each locus ranged from seven (*cfxE*) to eleven (*hemH* and *leuB*), with a mean of 9.14 alleles per locus. Analysis of 34 isolates of *S*. *haemolyticus* and ATCC29970 showed 24 new STs designated as ST18 to ST41 and three previously reported STs comprising ST1, ST3, and ST9 indicating heterogeneity among them. All the 34 isolates along with ATCC29970 were clustered into one major cluster, two minor clusters and eight singletons (Fig 2A). The major cluster consisted of isolates belonging to 15 STs comprising ST1, ST3, ST9, ST19, ST20, ST25, ST28, ST29, ST30, ST31, ST35, ST37, ST38, ST39, and ST40. The isolates from different sources clustered together showing identical STs, belonging to ST1, ST3, ST9, ST20, and ST21. Also, the isolates from the infected eyes and healthy conjunctivae showed identical STs, belonging to ST1 and ST20.

Table 1 shows allelic profiles identified till date among *S*. *haemolyticus* including 24 new allelic profiles. ST1 comprised of the largest number of isolates of which 14 out of 18 isolates were methicillin-resistant. All the STs, except four STs, comprising ST1, ST3, ST8, and ST9 belong to both methicillin-resistant and methicillin-sensitive isolates. Another MST considering all the previously reported STs and new proposed STs was also generated [3]. Upon comparison, all the STs clustered into one primary cluster, two minor clusters and eight singletons (Fig 2B). Cluster 1 comprised of 22 STs belonging to ST1, ST2, ST3, ST4, ST8, ST9, ST10, ST13, ST14, ST15, ST19, ST20, ST25, ST28, ST29, ST30, ST31, ST35, ST37, ST38, ST39, and ST40, respectively. Of the two minor clusters; cluster 2 comprised of 3 STs namely ST17, ST21, and ST26 and cluster 3 consisted of 2 STs namely ST23 and ST27. Fourteen STs were defined as singleton consisting of ST5, ST6, ST7, ST11, ST12, ST16, ST18, ST22, ST24, ST32, ST33, ST34, ST36, and ST41. MST when generated using all the STs showed that both new and previously reported STs could group into cluster 1 and cluster 2 (Fig 2B).

**Table 1:** Allelic profiles of sequence types (STs) of *S*. *haemolyticus* strains isolated from a variety of infections and also included previously reported STs (ST1-ST17).

Sequence Type	No. of isolates (no. of methicillin resistant)	Allelic profile (allele no.)						
		*arc*	SH1200	*hemH*	*leuB*	SH1431	*cfxE*	*Ribose ABC*
1	13(12); **5(2)**	2	1	1	1	1	1	4
2	2(2)	1	1	1	1	1	1	1
3	8(6); **2(1)**	1	1	1	1	1	1	4
4	3(3)	1	1	1	1	2	2	2
5	1(0)	5	4	4	3	4	4	x
6	1(0)	4	3	3	2	3	3	5
7	1(0)	2	4	5	4	4	3	3
8	5(4)	1	5	1	1	2	1	4
9	2(2); **2(1)**	2	5	1	1	2	1	4
10	1(1)	1	5	1	1	1	1	1
11	1(1)	3	2	2	1	2	1	1
12	1(1)	1	5	2	5	6	1	4
13	2(2)	1	1	1	1	2	1	2
14	1(1)	1	5	2	1	2	1	4
15	1(1)	2	1	1	1	1	5	4
16	1(1)	2	1	1	1	1	2	2
17	1(1)	3	5	6	1	5	1	4
**18**	**1(0)**	**6**	**6**	**7**	**6**	**7**	**6**	**6**
**19**	**1(1)**	1	**7**	1	1	1	1	4
**20**	**2(2)**	3	1	1	1	1	1	**7**
**21**	**2(0)**	1	5	6	1	5	1	4
**22**	**1(1)**	1	5	1	1	5	**7**	4
**23**	**1(0)**	1	4	**8**	**7**	**8**	4	4
**24**	**1(0)**	**6**	**6**	**9**	**6**	**9**	**6**	**8**
**25**	**1(1)**	2	1	1	1	5	1	4
**26**	**1(0)**	1	5	6	1	5	1	1
**27**	**1(1)**	1	4	**8**	**7**	**8**	1	4
**28**	**1(1)**	3	1	1	1	1	1	1
**29**	**1(0)**	1	1	1	1	2	1	4
**30**	**1(1)**	2	5	1	1	1	1	4
**31**	**1(1)**	2	1	1	**8**	1	1	4
**32**	**1(1)**	1	1	1	**9**	5	1	4
**33**	**1(1)**	2	8	**10**	1	5	1	4
**34**	**1(1)**	2	1	1	**8**	5	1	9
**35**	**1(1)**	**7**	1	1	1	1	1	4
**36**	**1(0)**	1	5	1	**10**	5	1	4
**37**	**1(1)**	1	1	1	**11**	1	1	4
**38**	**1(1)**	2	1	1	1	1	1	**9**
**39**	**1(1)**	3	1	1	1	1	1	4
**40**	**1(1)**	1	1	1	1	1	7	4
**41**	**1(1)**	2	**9**	**11**	**8**	1	6	4

Bold: New sequence types (STs) and alleles.

ST1-ST17: STs and profiles were reported previously by Cavanagh et al. [3]

The data of this study thus suggest that MLST scheme can be widely used to study population structure and diversity among *S*. *haemolyticus* isolates. The developed MLST database could select STs and assign new STs among *S*. *haemolyticus* after adding new sequence information to the database.
